# Left sided Richter type obturator hernia causing intestinal obstruction: A case report

**DOI:** 10.1016/j.amsu.2018.10.011

**Published:** 2018-10-11

**Authors:** Kusay Ayoub, Nihad Mahli, M. Fateh Dabbagh, Bashar Banjah, Bassel Banjah

**Affiliations:** a Department of Surgery, Aleppo University Hospital, Aleppo, Syria; b Faculty of Medicine, University of Aleppo, Aleppo, Syria

**Keywords:** Obturator, Hernia, Intestinal obstruction, Richter, Case report, Incarcerated

## Abstract

**Introduction:**

Although relatively rare, an obturator hernia is a significant cause of intestinal obstruction. It usually occurs in emaciated elderly females. Computed tomography is the imaging modality of choice to diagnose obturator hernias.

**Case report:**

In this report we present a case of an elderly female who presented to the emergency department with features suggesting bowel obstruction. The patient was admitted to the hospital and was initially managed conservatively. Two days later the patient underwent an exploratory laparotomy and was diagnosed with a left sided Richter type obturator hernia. The hernia was successfully reduced and the necrotic bowel was resected with end to end anastomosis.

**Discussion:**

An obturator hernia is a rare type of abdominal hernias which often occurs in very thin old females. Patients with obturator hernias usually present with symptoms of acute or intermittent small bowel obstruction. Mild symptoms without abdominal pain may be due to incomplete obstruction or Richter type hernia. Computed tomography is considered the gold standard diagnostic modality for obturator hernias. An early surgical intervention is the treatment of choice.

**Conclusion:**

The clinical diagnosis of an obturator hernia is often difficult due to its nonspecific symptoms and infrequent signs. Yet early diagnosis is mandatory because its delay contributes to bowel necrosis and to the poor prognosis in these patients. Surgery remains the only effective management of this condition.

## Highlights

• Obturator hernias are a rare cause of intestinal obstruction.
• An obturator hernia usually occurs in very thin elderly females.
• Obturator hernias present with symptoms of intestinal obstruction.
• Computed tomography is the imaging modality of choice to diagnose obturator hernias.
• The only management of this condition is surgery through either laparotomy or laparoscopy.

## 1 Introduction

Obturator hernia is a rare clinical condition that may cause intestinal obstruction. It accounts for 0.5–1.4% [1] of all abdominal hernias and usually occurs in elderly emaciated multiparous women [2]. The diagnosis of this disease is often delayed because it is difficult to detect [1]. Unfortunately patients with delayed diagnosis often have high morbidity and mortality rates and most of them undergo partial intestinal resection [3]. Here we report a patient who presented to our emergency service with manifestations of bowel obstruction resulting from an incarcerated abdominal hernia.

### 1.1 Case presentation

**Fig. 1:**
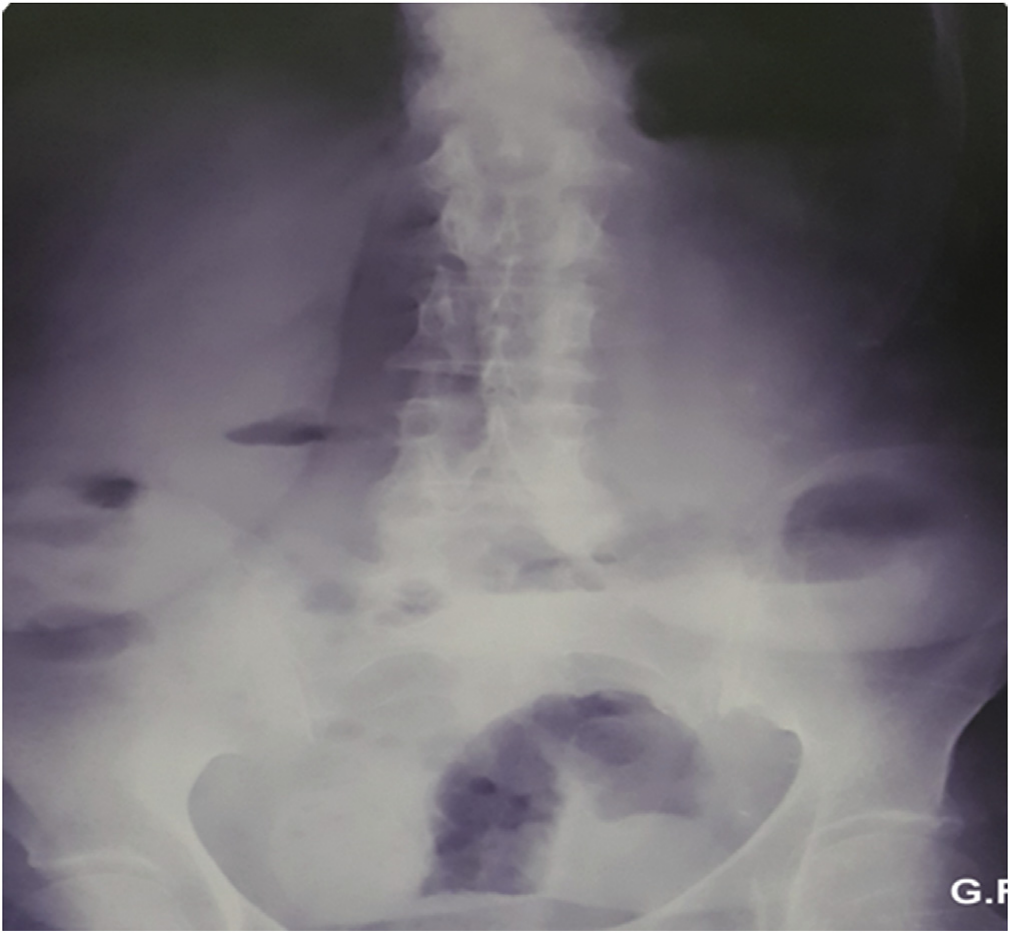
Plain Abdominal X-ray showing air-fluid levels with air in the rectum.

**Fig. 2:**
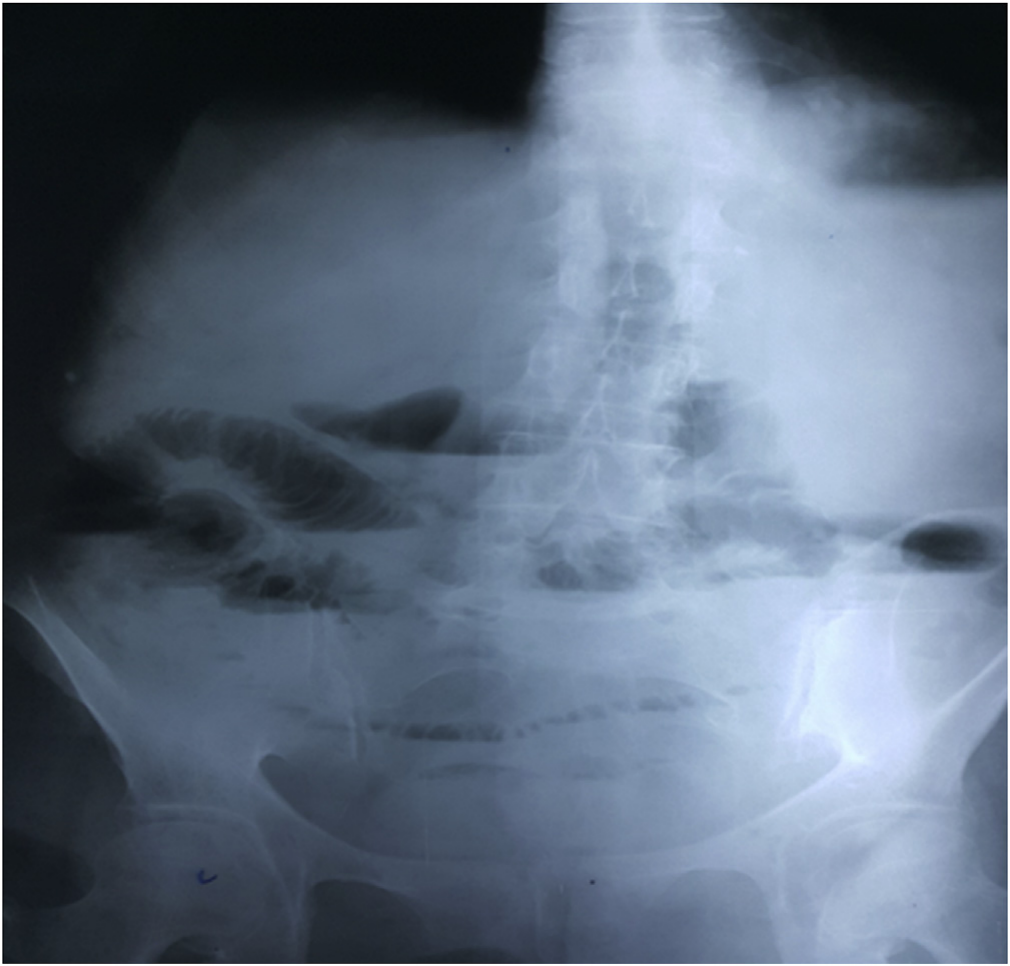
Plain abdominal X-ray showing increased air-fluid levels without air in the rectum.

**Fig. 3:**
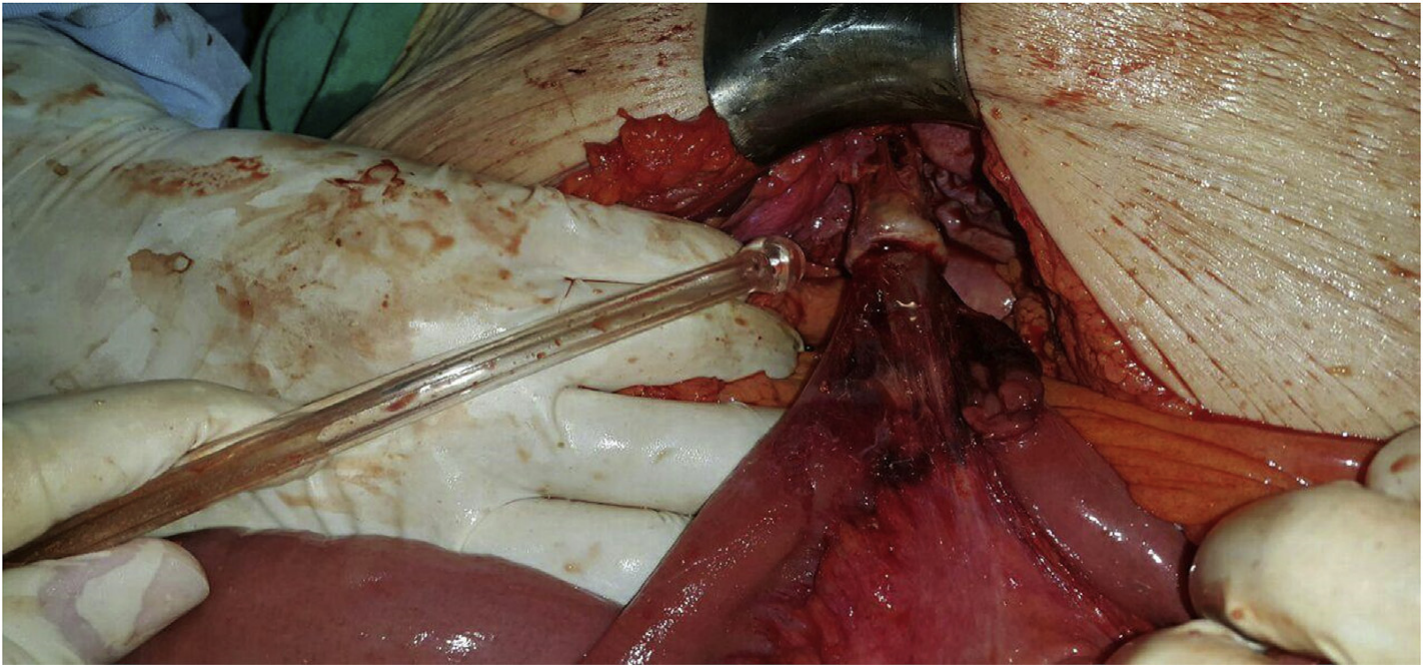
Intraoperative image showing a part of the bowel protruding through the left obturator foramen.

**Fig. 4:**
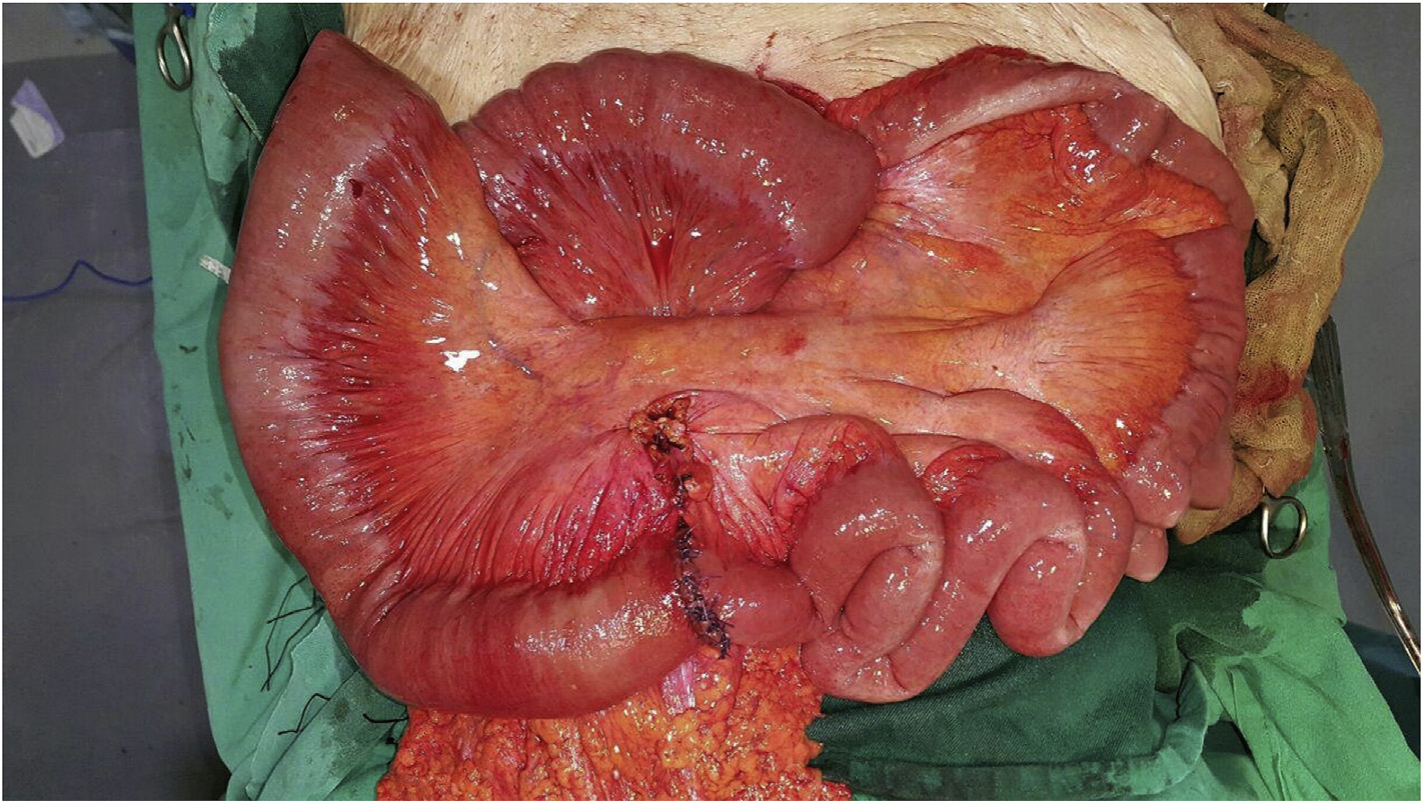
Intraoperative image showing bowel anastomosis after resection of the necrotic bowel.

An 83 year old female presented to the emergency department complaining of vomiting and constipation that started a few days ago. She did not report abdominal pain or distention. The patient was otherwise healthy and has not undergone any previous abdominal or pelvic surgeries. On arrival the patient was malnourished, well oriented, and afebrile. Her blood pressure was 100/80 mm hg and her pulse was 80 beats per minute. On physical examination the abdomen was soft, not tender, and not distended, and no hernias were palpable. The rectum was found to be empty on rectal examination. Laboratory findings showed a hemoglobin level of (17.6 gr/dL), mild leukocytosis (11400/μL) with a left shift (81%), and a platelets level of (279000/μL). Urea (285 mg/dL) and creatinine (3.9 mg/dL) levels were elevated. The patient also had low serum sodium (131 mEq/L) and a normal potassium level (3.5 mEq/L). The initial plain abdominal radiography showed air fluid levels with air present in the rectum suggesting small intestinal obstruction (Fig. 1). Abdominal ultrasonography revealed dilated small bowel loops. The patient was admitted to the hospital and was initially managed conservatively with nasogastric suction and intravenous fluids. The nephrology unit was consulted for the diagnosis of renal failure. Two days later the patient did not improve significantly. Her white blood cells rose to (12000/μl) but her kidney function improved. Plain abdominal radiography was repeated and it showed increased air fluid levels without air in the rectum which is also consistent with small intestinal obstruction (Fig. 2). Because the cause of small intestinal obstruction was unclear, the patient underwent an exploratory laparotomy via a midline incision. As the abdominal cavity was reached, it was possible to visualize a Richter type hernia protruding through the left obturator canal (Fig. 3). We reduced the hernia successfully and examined the bowel for signs of necrosis. A small necrotic area was found and was resected with end to end anastomosis (Fig. 4). The defect at the hernia site was closed by interrupted sutures. The patient had an uneventful recovery and no recurrence of the hernia was noted during follow up.

## 2 Discussion

An obturator hernia is a rare type of abdominal hernias, accounting for 0.5–1.4% of all hernias [1]. It was first reported by Arnaud de Ronsil in1724 [2] and was first successfully repaired by Henry Ombre in 1851 [2]. An obturator hernia often occurs in a very thin old female without previous surgeries and is sometimes referred to as ‘Little old Lady Hernia’ [2]. It is estimated that obturator hernias are nine times more common in females than males [3]. The wider pelvis, the greater transverse diameter of the obturator foramen, and the weakened pelvic tissues in females make them more susceptible to this hernia [4]. Like other hernias, any factor that increases intraabdominal pressure or weakens the hernia site (e.g. constipation, ascites, COPD, kyphoscoliosis, multiparity, and aging) predisposes the patient to this hernia. In addition, the loss of preperitoneal fat around the obturator nerves and vessels due to aging or, malnutrition makes the obturator canal wider and facilitates herniation [3]. Knowledge of the anatomy of the obturator canal is essential to understand the presentation and diagnosis of obturator hernias. The obturator foramen is formed by the pubic and ischial bones and is covered by the obturator membrane. In its anterior superior part, the obturator membrane is perforated by the obturator artery, vein, and nerve which travel between the internal and external obturator muscles. A hernia sac may develop through this defect and an organ, most commonly ileum and sometimes the omentum, protrudes into it [5,6]. Herniation often occurs in the right side because the sigmoid colon which covers the left obturator foramen may prevent herniation [7]. The development of an obturator hernia occurs in three stages [3]. In stage 1, the preperitoneal connective tissue and fat enter the obturator canal. In Stage 2, a peritoneal sac forms [3]. Stage 3 is characterized by symptoms due to incarceration of an organ [3]. Patients with obturator hernias usually present with symptoms of acute or intermittent small bowel obstruction due to incarceration of the intestine in the obturator canal. These may include nausea, vomiting, abdominal pain, and constipation. Mild symptoms without abdominal pain may be due to incomplete obstruction or Richter type hernia. In addition patients may complain of medial thigh pain (Howship Romberg sign) [8], or examination may show absent thigh adductor reflex in the presence of knee reflex (Hannington Kiff sign) due to compression of the obturator nerve [8]. The Howship Romberg sign is considered pathognomonic, but only occurs in 15–50% of cases [8]. The clinical diagnosis of an obturator hernia is difficult due to its nonspecific symptoms and infrequent signs. Plain abdominal radiography and abdominal ultrasonography usually reveal signs of intestinal obstruction. However, computed tomography is considered the gold standard diagnostic modality [7]. In this case we presented an underweight old female with symptoms of intestinal obstruction. Computed tomography was not done due to technical issues. The patient was diagnosed intraoperatively with a left sided Richter's type obturator hernia. Since the patient was symptomatic at presentation, her hernia was in the third stage of development. In general, obturator hernias are treated surgically. Conservative management may be sought when the cause of intestinal obstruction is uncertain or when the patient declines surgery, but it is usually associated with increased morbidity and mortality rates [3]. Several surgical approaches have been described in literature including abdominal, retro pubic, obturator, inguinal, and laparoscopic approach [3]. Although the laparoscopic approach is associated with fewer complications, the abdominal approach is often favored [9]. This approach allows the surgeon to confirm the diagnosis, avoid injuries to obturator nerve and vessels, and resect the bowel when necessary [9].The defect can be closed primarily with interrupted or continuous sutures, with the use of surrounding tissues like the omentum, or using a synthetic mesh [10].

## 3 Conclusion

Although the obturator hernia is a rare surgical case, it must be always kept in mind as a cause of small bowel obstruction, especially in thin elderly females. Early Diagnosis and intervention are essential to avoid complications like bowel necrosis. The only management of this condition is surgery through either laparotomy or laparoscopy.

## Consent

Informed consent was obtained from the patient for publication of this case report and accompanying images.

## Provenance and peer review

Not commissioned, externally peer reviewed.

## Ethical approval

Not applicable.

## Sources of funding

Authors did not receive any Funding.

## Author contribution

Kusay Ayoub and Nihad Mahli: revised the case.

M.Fateh Dabbagh: Case report writing and design.

Bashar Banjah: Subject Research.

Bassel Banjah: edited final draft.

## Conflicts of interest

No conflicts to declare.

## Research registration number

Not applicable.

## Trial registry number

This is a case report.

## Guarantor

Kusay Ayoub, Nihad Mahli.

## Appendix A Supplementary data

SCARE Guideline Checklist SCARE Guideline Checklist
